# Assigning disease clusters to people: A cohort study of the implications for understanding health outcomes in people with multiple long-term conditions

**DOI:** 10.1177/26335565241247430

**Published:** 2024-04-17

**Authors:** Thomas Beaney, Jonathan Clarke, David Salman, Thomas Woodcock, Azeem Majeed, Mauricio Barahona, Paul Aylin

**Affiliations:** 1 Department of Primary Care and Public Health, 4615 Imperial College London, London, UK; 2 Centre for Mathematics of Precision Healthcare, Department of Mathematics, 4615 Imperial College London, London, UK

**Keywords:** Multiple long-term conditions, clusters, multimorbidity, health outcomes, segmentation

## Abstract

**Background:**

Identifying clusters of co-occurring diseases may help characterise distinct phenotypes of Multiple Long-Term Conditions (MLTC). Understanding the associations of disease clusters with health-related outcomes requires a strategy to assign clusters to people, but it is unclear how the performance of strategies compare.

**Aims:**

First, to compare the performance of methods of assigning disease clusters to people at explaining mortality, emergency department attendances and hospital admissions over one year. Second, to identify the extent of variation in the associations with each outcome between and within clusters.

**Methods:**

We conducted a cohort study of primary care electronic health records in England, including adults with MLTC. Seven strategies were tested to assign patients to fifteen disease clusters representing 212 LTCs, identified from our previous work. We tested the performance of each strategy at explaining associations with the three outcomes over 1 year using logistic regression and compared to a strategy using the individual LTCs.

**Results:**

6,286,233 patients with MLTC were included. Of the seven strategies tested, a strategy assigning the count of conditions within each cluster performed best at explaining all three outcomes but was inferior to using information on the individual LTCs. There was a larger range of effect sizes for the individual LTCs within the same cluster than there was between the clusters.

**Conclusion:**

Strategies of assigning clusters of co-occurring diseases to people were less effective at explaining health-related outcomes than a person’s individual diseases. Furthermore, clusters did not represent consistent relationships of the LTCs within them, which might limit their application in clinical research.

## Introduction

Multimorbidity, or Multiple Long-Term Conditions (MLTC) is a common and growing phenomenon worldwide, associated with increased mortality,^1^ poorer quality of life,^2^ increased healthcare use,^3,4^ and difficulties in accessing and navigating the healthcare system. MLTC is defined as the co-occurrence of two or more long-term conditions (LTCs) in one person, but is a crude marker of medical complexity. Hence, there is growing interest in defining groupings or ‘clusters’ of similar diseases representing distinct phenotypes. It is hoped that characterisation of clusters will enable identification of shared causes of diseases, tailoring of preventive and therapeutic strategies and the development of more integrated health services.^5,6^ Many studies have been conducted over the last decade to identify clusters of LTCs that tend to co-occur, with clusters of cardio-metabolic and mental health conditions the most replicable.^7,8^

Despite growing understanding of which LTCs tend to co-occur, it remains unclear how we can best use these disease clusters to understand or predict disease determinants and health outcomes. Diseases do not exert effects in isolation but are shaped by complex bio-psycho-social factors specific to a person. People, however, may not fall neatly into one disease cluster, instead having diseases spanning multiple clusters. This requires a strategy to assign clusters to people, of which several approaches have been applied before. These range from assigning a cluster if a person has one or more,^9^ two or more,^10–12^ or three or more^13^ diseases in the cluster, or if a person has at least half of their conditions in one cluster.^14,15^ An alternative, which we propose here, is to represent a person by the number or proportion of LTCs they have in each disease cluster, accounting for the full distribution of diseases across clusters.

The relative effectiveness and limitations of these strategies are unclear, with a lack of empirical evidence to guide researchers in their choice of method. To address this gap, we aim to directly compare a range of strategies to assign disease clusters to people and test how well each performs at explaining health-related outcomes. We do this using a set of disease clusters identified previously by our team using natural language processing (NLP) and graph-based clustering applied to a representative sample of ten million people with MLTC in England.^16^ First, we compare the distributions of people amongst clusters using the different strategies, including the percentage of non-assignment and multiple assignment of people to clusters. Second, we compare how well each strategy performs at explaining 1-year mortality, emergency department (ED) attendance, and emergency hospitalisation. Third, for the best-performing clustering strategy, we compare variation in age at onset and in the three outcomes between clusters and assess the consistency of associations between diseases within the same cluster.

## Methods

### Data

We conducted a cohort study using data from the Clinical Practice Research Datalink (CPRD) Aurum dataset, collected from General Practice (GP) electronic healthcare records (EHRs) in England.^17^ We included all patients aged 18 years or over who were defined by CPRD as ‘research acceptable’ (a quality indicator such as having a valid registration date and date of birth)^18^, were registered to a GP practice on 1^st^ January 2015 and had two or more LTCs (see definition below). We also included only those eligible for linkage to secondary care data (98.8% of patients) and those registered to a GP practice for at least one year (90.8% of patients) to ensure sufficient time for data input (see appendix Figure A1).^19^ Demographic data include age, gender and ethnicity (see appendix p.2 for cleaning rules). The 2019 Index of Multiple Deprivation (IMD) of a patient’s area of residence was used as a marker of socio-economic deprivation, where 1 indicates the least deprived decile, and 10 the most deprived.^20^

Secondary care data was sourced from Hospital Episode Statistics (HES) and data on death registrations from the Office for National Statistics, both linked to the Aurum dataset by CPRD using a pseudonymised identifier.^21^ As CPRD Aurum data also includes a marker of mortality, where there were differences in the dates between sources, we reconciled these using the algorithm recommended by Delmestri and Prieto-Alhambra (2020) (see appendix p.2).^22^

### LTCs and cluster definitions

We used the assignment of 212 LTCs to 15 clusters identified in our previous study, representing clusters of diseases that tend to occur together in sequence.^16^ In brief, in our earlier work, we tested different word embedding models popular in NLP. Applied to text data, these methods generate a quantifiable vector representation of words (known as a vector embedding) by learning the context that words appear in a sequence, such as a sentence or document. In our case, we interpret the order of diseases developed over time in a person as analogous to the order of words in a sentence and used the algorithms to generate a vector embedding for each disease. We applied these methods to the time-ordered sequences of diagnostic codes in the EHR representing 212 LTCs for 10.5 million patients registered between 2015 and 2020 in CPRD. We found that the best-performing NLP method was better at explaining well-established disease associations than a method which used only co-occurrence (multiple correspondence analysis).^16^ Using the disease representations, we calculated the cosine similarity of each disease vector embedding to each other, and used a graph-based clustering algorithm (Markov Multiscale Community Detection) to identify groupings of similar diseases with 25, 15 and 7 clusters, which were clinically interpretable. Here, we select the 15-cluster resolution, striking a balance between granularity and interpretability, but conduct a sensitivity analysis using the 25-cluster resolution. The full list of 212 LTCs and assignment to the 15 clusters are shown in the appendix (p.3-5) and an evaluation of their interpretability is described in our previous paper.^16^

### Mapping disease clusters to people

We compared the following five strategies to assign disease clusters to people, using a binary classification (1 if the cluster is assigned, and 0 otherwise):

1. One or more: assigning a cluster if a person has at least one disease in the cluster.
2. Two or more: assigning a cluster if a person has at least two diseases in the cluster.
3. Three or more: assigning a cluster if a person has at least three diseases in the cluster.
4. Modal: assigning the (one) cluster in which the modal number of diseases fall. In the case of ties, where two or more clusters had the same number of diseases, the modal cluster assignment was selected at random from amongst the tied clusters.
5. Majority: assigning to a cluster if over 50% of a person’s diseases are within the cluster.

We also compared two other strategies, that instead represented the count or proportion of a person’s diseases that fall into each cluster:

1. Count: assigning the number of diseases a person has within each cluster.
2. Proportional: assigning the proportion of a person’s diseases within each cluster.

**Figure 1.**
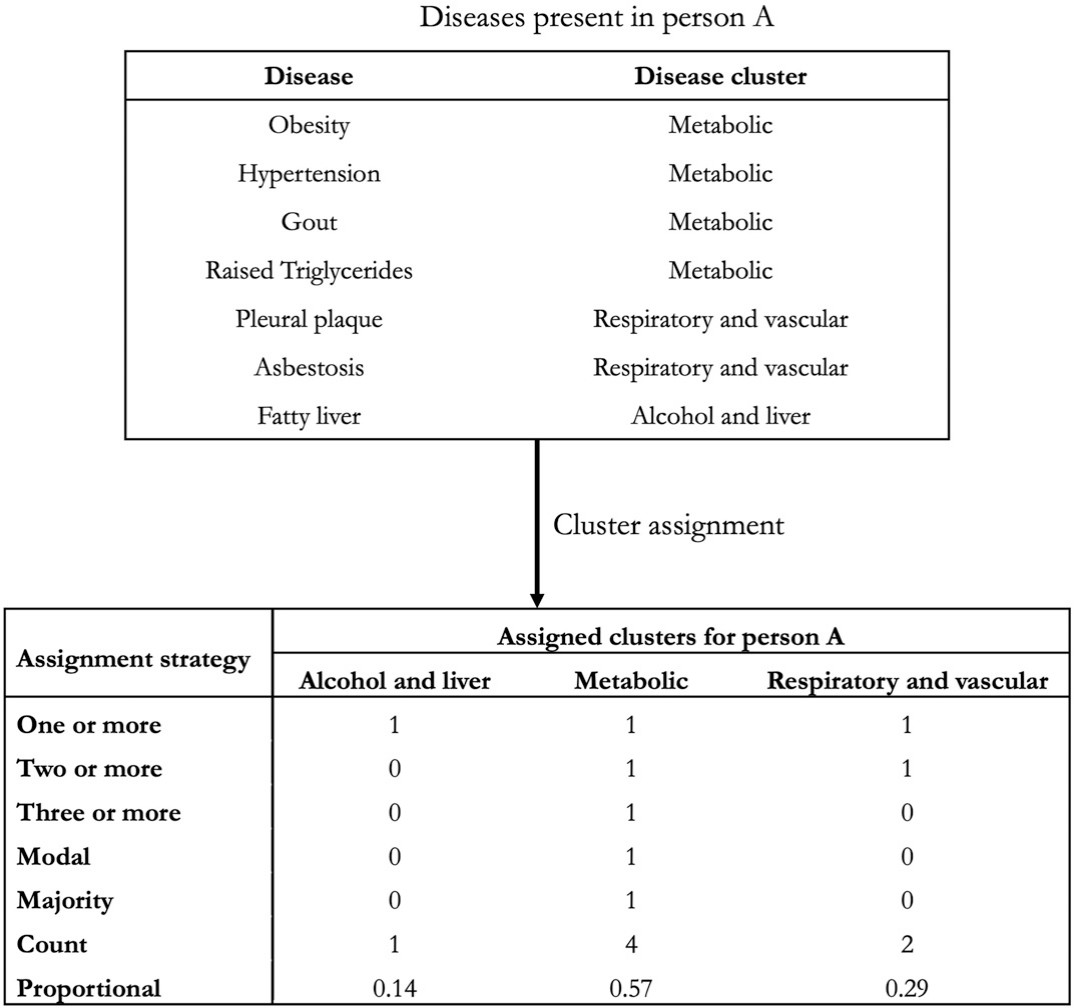
Hypothetical distribution of seven LTCs to three clusters in one person and the resulting cluster assignment under each strategy. Note: In this example, person A has four diseases in the metabolic cluster, two in the respiratory and vascular cluster and one in the alcohol and liver cluster. Requiring one or more diseases in a cluster results in assignment of all three clusters, requiring two or more results in assignment of two clusters, and requiring three or more results in assignment of one cluster. Under the modal and majority strategy, only the metabolic cluster is assigned. The count strategy retains the number of diseases within each cluster, and the proportional method accounts for the relative distribution of diseases across each cluster.

An example of the assignment for a hypothetical person with seven LTCs spanning three clusters is shown in Figure 1.

### Statistical analysis

For all patients registered to a GP practice on 1^st^ January 2015, we identified their set of existing LTCs based on diagnostic codes recorded before 2015. For each of the cluster assignment strategies, we calculated the percentage of patients that were not assigned to any cluster, the percentage assigned to only one, and the percentage assigned to more than one cluster. We also calculated the percentage of patients who were assigned to clusters such that this represented all their LTCs, and the median (and interquartile range (IQR)) number of LTCs for each patient that were not assigned a cluster.

We determined the date of diagnosis for each LTC based on the earliest observation date recorded for that condition. We used this to calculate the median age at diagnosis for each disease, and for each cluster, calculated the median age at diagnosis of any disease in the cluster.

We compared the performance of the seven strategies at explaining three outcomes, occurring over 1-year of follow-up (i.e., during 2015 only):

1. Death from any cause
2. Any ED attendance
3. Any emergency hospital admission

To ensure equal follow-up periods, we excluded 305,142 (4.9%) patients who de-registered in the follow-up period (i.e., if they moved to a GP practice which is not part of the study dataset). To examine associations, we constructed separate logistic regression models for each outcome variable and included the fifteen clusters as independent predictors. The clusters were modelled as binary variables, except for the count and proportional methods, where clusters were modelled as continuous variables. Age, gender, ethnicity and IMD decile were included in all models as covariates. We also constructed a separate model inputting the individual diseases (N=212) as binary independent variables, rather than clusters, as a comparison to a disease-only approach. Model performance was compared using the Akaike Information Criterion (AIC), where a lower value indicates a better-fitting model that explains a greater amount of variability, accounting for the number of parameters in the model (i.e., where two models explain the same amount of variability, the model with fewer parameters will have a lower AIC). In our case, given each cluster strategy has the same number of input variables, a lower AIC indicates greater explained variance. The statistical equations for the models are given in the appendix (p.6). To assess the sensitivity of the best-performing strategy to the number of clusters, we conducted a sensitivity analysis running the logistic regression models for each outcome instead using the 25-cluster resolution.

## Results

A total of 6,286,233 patients aged ≥18 years and with MLTC were included (see appendix Figure A1). The mean age was 53 (SD 18) years, with more females (53.1%) than males (46.9%) (Table 1). Most patients (86.2%) were of White ethnicity and there were relatively more patients living in less deprived areas (52.3% in the five least deprived IMD deciles). The median number of LTCs per patient was eight (IQR: 5 – 11). There was a trend towards higher numbers of LTCs at higher ages, and in White and South Asian ethnicities in comparison to other ethnicities, with no strong differences between genders or with socioeconomic deprivation.

**Table 1.** Patient characteristics and number of LTCs and clusters per patient.

Patient characteristic	Total	Percent	Median (IQR) number of LTCs
Age (years)			
18-29	707208	11.3%	3 (2, 5)
30-39	803366	12.8%	4 (3, 6)
40-49	1108696	17.6%	6 (4, 8)
50-59	1225401	19.5%	7 (5, 10)
60-69	1081293	17.2%	9 (6, 12)
70-79	797941	12.7%	10 (7, 14)
80+	562328	8.9%	12 (9, 16)
Gender			
Female	3339743	53.1%	8 (5, 12)
Indeterminate	58	<0.1%	8 (5, 12)
Male	2946432	46.9%	8 (5, 11)
Ethnicity			
White	5419210	86.2%	8 (5, 12)
South Asian	372336	5.9%	8 (5, 11)
Black	220687	3.5%	7 (4, 10)
Other	83472	1.3%	6 (4, 9)
Mixed	64109	1.0%	6 (4, 9)
Missing	126419	2.0%	4 (3, 6)
IMD decile			
1 (least deprived)	710805	11.3%	8 (5, 11)
2	652898	10.4%	8 (5, 11)
3	665105	10.6%	8 (5, 11)
4	662155	10.5%	8 (5, 11)
5	594419	9.5%	8 (5, 11)
6	624994	9.9%	8 (5, 12)
7	622678	9.9%	8 (5, 12)
8	598585	9.5%	8 (5, 12)
9	598129	9.5%	8 (5, 12)
10 (most deprived)	552291	8.8%	8 (5, 12)
Missing	5174	0.1%	8 (5, 11)
**Total**	**6286233**		**8 (5, 11)**

### Assignment of disease clusters to people

We compared seven strategies to assign disease clusters to people. The one or more, count and proportional methods produced the same distributions of clusters amongst people, with all patients assigned at least one cluster, 90.7% assigned more than one, and with the clusters representing all the LTCs for each person (Table 2). The percentage of non-assignment increased from 17.3% to 51.9% if requiring two or more, or three or more LTCs in a cluster, respectively, with both cases resulting in few patients for whom the clusters represented all their LTCs (17.0% and 3.7%, respectively). The modal and majority methods resulted in a maximum of one cluster assigned, but with few patients having all their diseases represented by a cluster (9.3% for both). Applying the modal method, for 2,050,150 (32.6%) patients, there was a tie for the modal cluster assignment (for which one of the tied clusters was then selected at random).

**Table 2.** Assignment patterns comparing seven strategies assigning clusters to people.

Cluster assignment strategy	Number (%) with no clusters assigned	Number (%) with only one cluster assigned	Number (%) with more than one cluster assigned	Number (%) with all LTCs assigned to a cluster	Median (IQR) number of LTCs per person not assigned in a cluster
One or more	0 (0)	585,164 (9.3)	5,710,069 (90.7%)	6,286,233 (100)	0 (0)
Two or more	1,087,653 (17.3)	2,429,950 (38.7)	2,768,630 (44.1)	1,068,906 (17.0)	2 (1-3)
Three or more	3,262,466 (51.9)	2,030,199 (32.3)	993,568 (15.8)	231,957 (3.7)	3 (2-5)
Modal	0 (0)	6,286,233 (100)	0 (0)	585,164 (9.3)	2 (1-5)
Majority	4,235,330 (67.4)	2,050,903 (32.6)	0 (0)	585,164 (9.3)	4 (2 -7)
Count *	0 (0)	585,164 (9.3)	5,710,069 (90.7%)	6,286,233 (100)	0 (0)
Proportional *	0 (0)	585,164 (9.3)	5,710,069 (90.7%)	6,286,233 (100)	0 (0)

*For count and proportional strategies, assignment determined by any non-zero value, and gives identical distribution to ‘One or more’.

### Associations with mortality, ED attendances and hospitalisation

We compared the performance of the seven clustering strategies at explaining mortality, any ED attendance and any emergency admission over 1 year, in the 5,981,091 (95.1%) patients who had not de-registered over the study period (Table 3). We also compared these strategies with using knowledge of the individual diseases as inputs, and found this performed better than all of the clustering strategies for all three outcomes, indicated by the lowest AIC. Of the clustering strategies, the count method performed best for all outcomes and the majority method performed worst, followed by the modal method of assignment. There were relatively greater differences between strategies in explaining the associations with mortality, than for ED attendance or emergency admissions, suggesting that choice of strategy has a larger impact for mortality than the other two outcomes. In a sensitivity analysis using 25 clusters rather than 15 for the count strategy, AIC values were slightly lower, but performance of the clusters remained substantially worse than using individual diseases (appendix Table A1).

**Table 3.** AIC values from multivariable logistic regression models for mortality, ED attendance and emergency admissions over 1 year, for different cluster assignment strategies.

Cluster assignment strategy	Mortality	ED attendances	Emergency admissions
One or more	681376.7	5830342	3023973
Two or more	691359.8	5863727	3058880
Three or more	703642.9	5887997	3086940
Modal	705865.9	5931060	3123758
Majority	711103.1	5921181	3120128
Count	**672196.4**	**5817671**	**3008956**
Proportional	678660.7	5901948	3076305
Diseases*	638663.6	5794989	2975097

* Diseases presented for comparison, and uses the individual diseases as binary inputs, rather than the clusters. Values in bold are the lowest AIC values (indicating better performance) for each outcome, amongst the seven cluster strategies.

### Variation between and within clusters

**Figure 2.**
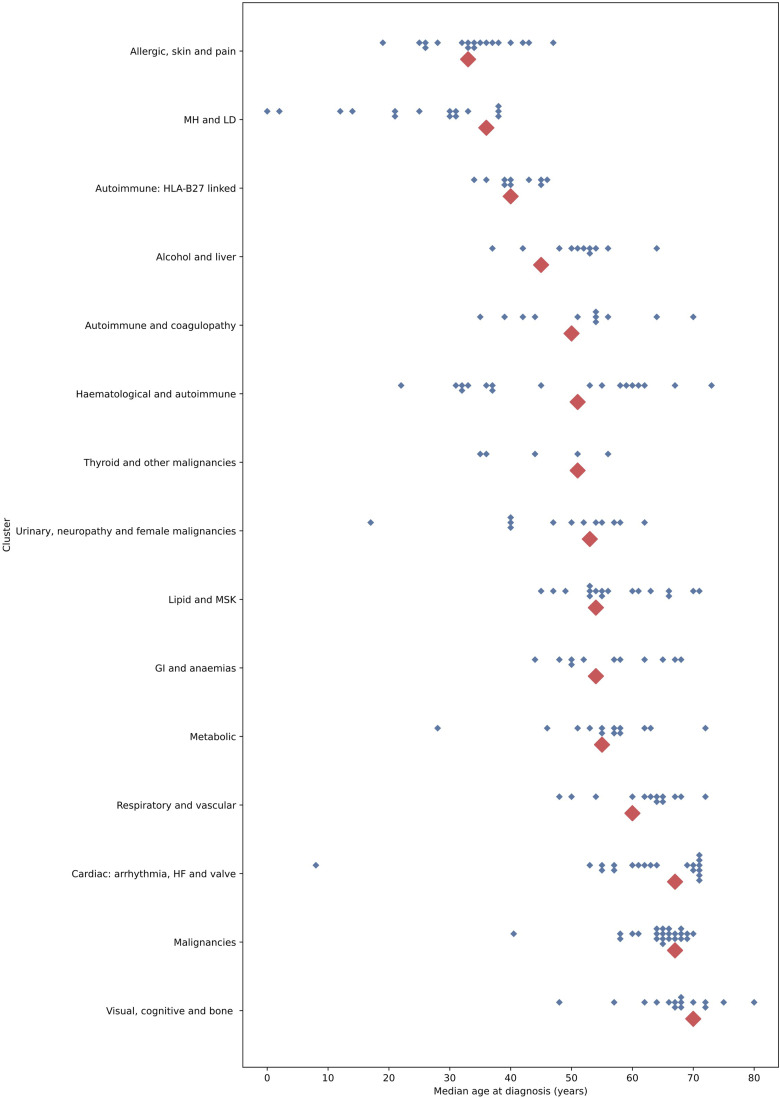
Median age at diagnosis between and within clusters, using the count assignment strategy. Note: blue diamonds represent median age at diagnosis of each of the 212 diseases, categorised according to their assignment to one of fifteen clusters shown on the y axis. Larger red diamonds represent the median age at diagnosis for the whole cluster. GI = Gastrointestinal; HF = Heart Failure; LD = Learning Disabilities; MH = Mental Health; MSK = Musculoskeletal.

For the best performing count strategy, we compared variation between and within the clusters in age at diagnosis and associations with outcomes. There were substantial differences in the age at diagnosis between clusters, from a median of 33 years in the ‘allergic, skin and pain’ cluster to 70 years in the ‘visual, cognitive and bone’ cluster (Figure 2). Within clusters, there was substantial variation in the median age at diagnosis of each individual LTC (Figure 2). For the ‘mental health and learning disability’ cluster, the median age at diagnosis for the cluster was 36 years, but four of the individual conditions had a median age of diagnosis under 18 years.

**Figure 3.**
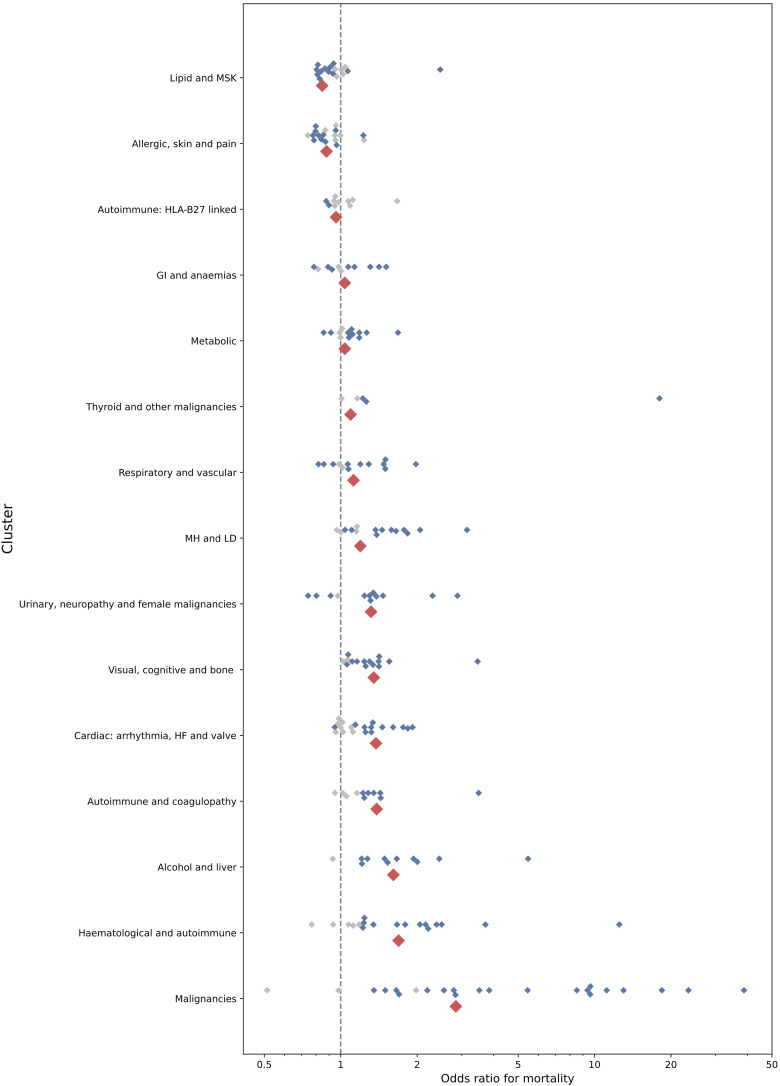
Odds ratios for 1-year mortality between and within clusters, from multivariable logistic regression, using the count assignment strategy. Note: smaller diamonds represent point estimates for each of the 212 diseases, categorised according to their assignment to one of fifteen clusters (shown on the y axis) from the disease regression model; blue represent statistically significant, and grey represent non-significant estimates. Larger red diamonds represent the point estimates for clusters from the cluster regression model (all statistically significant). Estimates, p-values and confidence intervals for clusters are given in the appendix Table A2. GI = Gastrointestinal; HF = Heart Failure; LD = Learning Disabilities; MH = Mental Health; MSK = Musculoskeletal.

People with conditions in the ‘malignancies’ cluster had the highest odds of mortality (Figure 3), ED attendance (Figure A2) and emergency admission (Figure A3) within one year, compared to others with MLTC without conditions in these clusters, after adjustment for age, gender, ethnicity and deprivation. People with conditions in the ‘allergic, skin and pain’ and ‘lipid and musculoskeletal’ clusters had a lower adjusted odds (aOR) of mortality within one year compared to others with MLTC without conditions in these clusters, but with greater ED attendances, and only small differences in the aOR of admission.

Within clusters, we found substantial variation in the range of associations of the individual diseases from the disease regression model (Figures 3, Figure A2and Figure A3). The median range of aORs for diseases within clusters was larger than the range of aORs between clusters (appendix table A5). This was particularly marked for the ‘malignancies’ cluster, in which, of statistically significant associations, primary malignancy of the prostate was associated with a 1.35 times increased adjusted odds of mortality, and mesothelioma was associated with a 38.8 times increased adjusted odds of mortality. For some clusters, the distribution in the range of aORs was substantially narrower, but most clusters included conditions representing non-significant associations, significantly lower (aORs<1) and significantly higher odds (aORs>1). For ED attendances and emergency hospital admissions, the range of values represented by individual conditions within a cluster tended to be narrower, but there remained substantial variation of the conditions within-cluster, particularly for the ‘Malignancies’ cluster.

## Discussion

In this study, we compared seven strategies to assign disease clusters to a cohort of people with MLTC in England. We found considerable differences between strategies in the number of clusters assigned per person, the number of people not assigned to any cluster and the number of a person’s LTCs not accounted for by a cluster. A strategy assigning the count of LTCs within a cluster performed best at explaining mortality, ED attendance and emergency hospitalisation, but still performed worse than using information on the single diseases, highlighting the loss of predictive information when aggregating diseases into clusters. There were also a larger range of effect sizes for individual LTCs within clusters than there were between clusters, demonstrating that disease clusters may not represent consistent patterns of their diseases with respect to outcomes.

Direct comparison with other studies is challenging due to differences in the number and composition of LTCs included, and the assignment of diseases to clusters, but to our knowledge, this is the first study to directly compare strategies to assign disease clusters to patients, and to assess the variability in associations both between and within clusters. We used a set of 212 LTCs, which will lead to a higher prevalence of MLTC than in many other studies, which have tended to include a smaller number of LTCs.^23^ Based on this broad set of LTCs, there were relatively fewer people with MLTC living in areas of greater deprivation, in contrast to some earlier studies using a smaller number of LTCs,^24^ but consistent with a recent study using the same data source and number of LTCs.^25^ We used the cluster assignment identified in our previous work,^16^ which represents clinically meaningful patterns, demonstrating strong links between cardiometabolic conditions and between mental health conditions, consistent with results from other studies.^8^ Although the absolute associations with health outcomes will vary according to the set of LTCs and assignment of LTCs to clusters, the relative differences between assignment strategies found here are likely to be generalisable.

### Implications for clustering in MLTC research

Our findings have implications for how disease clusters are used for both predictive applications and epidemiological research. The clusters used here were derived by metrics calculated from the occurrence of diseases in sequence with other diseases, and so they may not necessarily produce clusters of diseases similar with respect to other metrics, such as mortality or use of health services. Nevertheless, to be practically useful, a better set of clusters should be one that better explains variation in health-related outcomes.

All seven assignment strategies explained less variation in the three outcomes than when using information on the presence or absence of diseases in a person. This indicates that for applications such as predicting health outcomes, there is likely to be a loss of accuracy if using disease cluster assignment rather than diseases. In a sensitivity analysis, we found that use of 25 clusters performed slightly better than 15 clusters, but was still substantially worse than using individual diseases. There may be times when clustering as a form of dimensionality reduction is required, for example if using EHR data that includes thousands of diseases or clinical codes, to reduce the number of inputs. In this case, the count assignment strategy may be preferable, balancing a smaller number of dimensions with maximising the information retained for each person compared to other strategies. Interestingly, the count strategy performed better than the proportional method, highlighting that the absolute burden of diseases, and not only the relative composition of diseases across clusters, is important. However, researchers should consider that any of these methods are likely to worsen model performance and should test against inclusion of information on individual LTCs where possible. Hybrid approaches, using individual diseases for common conditions, and clusters for rare conditions, may represent a pragmatic balance and represents an avenue for further research.

In cases where disease clusters are to be used to explain associations with outcomes, rather than for prediction, loss of predictive power may be less critical. However, we found considerable variability in the associations of LTCs within the same cluster with age, mortality and healthcare utilisation, indicating that disease clusters may not represent homogenous categories with respect to determinants and outcomes. Although this may be expected given the clusters were derived by measures of disease co-occurrence, it highlights empirically that ascribing cluster-level associations to the individual diseases or people within a cluster may give spurious inferences, which could lead to bias and limit their practical use. We recommend that when investigating associations with disease clusters, researchers should assess the extent of variation within clusters and interpret cluster-wide associations cautiously.

An alternative approach to clustering diseases and then assigning to people, is instead to directly cluster people. This is analogous to ‘population segmentation’ approaches, which aim to risk stratify populations, often for the purposes of identifying individuals suitable for intervention.^26,27^ To generate a measure of similarity between people, these methods commonly incorporate information not only on diseases, but on demographics and healthcare utilisation.^26,27^ By including the relevant outcomes in the similarity measure, the derived clusters may be more meaningful with respect to those outcomes than similarity measures based only on disease co-occurrence, as used here. Recently, Huang *et al* (2021) implemented a combined approach to generate clusters of people, using information on both the co-occurrence of diseases, and similarities of the clusters with respect to predicting clinical endpoints, which is a promising avenue for future work.^28^

### Strengths and limitations

A strength of our study is the use of a large sample of patients with MLTC from a primary care data source representative of the population in England.^17^ EHRs contain real-world data collected during healthcare encounters and so are less prone to the selection biases common in prospective studies which can limit the generalisability to under-represented groups. Nevertheless, data are not collected primarily for research purposes and can have missing or incorrect data. Information on LTCs was based on coded information in the EHR, which may omit some diagnoses given most GP consultations are recorded as free-text,^29^ with greater coding rates for conditions which have financial incentives attached to coding.^30^

We tested several strategies to assign disease clusters to people, as described by others applied to MLTC research, but this is not intended to provide a comprehensive set of all possible strategies. For example, our clusters were derived from vector embeddings of the diseases, which could instead be averaged across a person to generate vector representations of people. Different strategies can be applied to assign a person vector to a cluster, such as assigning to the nearest cluster centroid, but would require different methodological assessment outside the scope of this paper. In modelling associations, to ensure a fair comparison between strategies, we did not consider interactions between the clusters or between sociodemographic covariates with the clusters, the significance of which are likely to differ between the assignment strategies. However, it is likely that allowing for interactions would improve the explanatory power of the clustering representations, highlighting potential synergistic effects of having conditions in more than one cluster, which could be an area for future research. We also considered a relatively short time period of 1 year when assessing outcomes. Although evaluation over longer time periods may impact on the absolute performance of the strategies, we would expect the relative differences between strategies to remain the same.

### Conclusion

Identification of clusters of co-occurring diseases are a key part of disentangling the complexity of MLTC and characterising distinct phenotypes. Our study, using real-world data, highlights the challenges of mapping disease clusters to people when seeking to understand health-related outcomes. Strategies which maximize the amount of information retained for each person performed better, but still resulted in worse explanatory power than using information on the individual diseases, highlighting the limitations of disease clusters in predictive applications. There was also considerable variability between the individual LTCs within a cluster with respect to outcomes, indicating that clusters may not represent uniform patterns, which could impact their meaningfulness in research and their implementation in clinical settings.

## Supplemental Material

Supplemental Material - Assigning disease clusters to people: A cohort study of the implications for understanding health outcomes in people with multiple long-term conditions Supplemental Material for Assigning disease clusters to people: A cohort study of the implications for understanding health outcomes in people with multiple long-term conditions by Thomas Beaney, Jonathan Clarke, David Salman, Thomas Woodcock, Azeem Majeed, Mauricio Barahona, and Paul Aylin in Journal of Multimorbidity and Comorbidity
