# Supplementary material for: Assigning disease clusters to people: A cohort study of the implications for understanding health outcomes in people with multiple long-term conditions
Source: J Multimorb Comorb. 2024 Apr 17;14:26335565241247430. doi: 10.1177/26335565241247430 (PMC11025432; doi:10.1177/26335565241247430)
Supplement: Supplemental Material - Assigning disease clusters to people: A cohort study of the implications for understanding health outcomes in people with multiple long-term conditions [file sj-pdf-1-cob-10.1177_26335565241247430.pdf]

Appendix: Assigning disease clusters to people: a cohort study of the implications for understanding health outcomes in people with multiple long-term conditions

Thomas Beaney[1,2], Jonathan Clarke[2], David Salman,[1] Thomas Woodcock[1], Azeem Majeed[1], Mauricio Barahona[2], Paul Aylin[1]

1. Department of Primary Care and Public Health, Imperial College London, London, United Kingdom
2. Centre for Mathematics of Precision Healthcare, Department of Mathematics, Imperial College London, London, United Kingdom

Corresponding Author:
Dr Thomas Beaney
Department of Primary Care and Public Health, Imperial College London, London, W6 8RP, United Kingdom
Email: thomas.beaney@imperial.ac.uk

Ethnicity data cleaning

NHS healthcare data in England uses the same ethnic categories as the 2001 census, which categorise ethnicity into a 5-level and a 16-level grouping.[1] We used the 5-level grouping using the ethnicity codes developed by Davidson *et al* (2021).[2] We removed four codes from these list representing examination findings, as suggesting clinician observed findings rather than reported by the patient. We followed the algorithm from Mathur *et al* (2020) to identify ethnicity:[3]

1. Remove duplicate ethnicity codes recorded on the same date.
2. Assign each code to the 5 and 16 level ethnicity categories.
3. If only one ethnicity code, use single code to define ethnicity.
4. If more than one code, use most frequently occurring ethnicity code.
5. If more than one code with the same count, use the latest ethnicity code instead.

Reconciliation of death recording

Deaths are recorded in both CPRD and in the Office for National Statistics (ONS) mortality statistics, of which the latter are generally regarded as the gold standard for recording deaths, given the legal requirement for its reporting in England and Wales.[4] However, the ONS figures represent only deaths recorded in England and Wales; the 7,524 deaths present only in CPRD may therefore reflect deaths that occurred outside of England and Wales, which would not be recorded in ONS but still represent genuine data. Delmestri and Prieto-Alhambra (2020) developed guidelines for reconciling the two sources of deaths.[5] Although their study used CPRD GOLD, the process of death registration in GP practices contributing to CPRD Aurum is likely to be similar, making these recommendations applicable. We adapted their recommendations to reconcile the two sources of death, as follows:

1. Use CPRD date of death if this is the only source present.
2. Use ONS date of death if this is the only source present and the match rank equals 1 or 2.
3. If data are recorded in both sources, use ONS only if match rank equals 1 or 2, or CPRD if match rank 3 or more.

Here, the match rank refers to CPRD's linkage criterion, with 1 and 2 corresponding to a match including the full date of birth to increase the robustness of the linkage.

Assignment of 212 LTCs to fifteen disease clusters:

Cardiac: arrhythmia, HF and valve	Malignancies
Atrial Fibrillation<br>Heart failure<br>Nonrheumatic aortic valve disorders<br>Supraventricular tachycardia<br>Nonrheumatic mitral valve disorders<br>Cardiomyopathy: other<br>Pleural effusion<br>Congenital Septal Defect<br>Right bundle branch block combinations<br>Left bundle branch block<br>Dilated cardiomyopathy<br>Hypertrophic Cardiomyopathy<br>Atrioventricular blocks<br>Ventricular tachycardia<br>Rheumatic Valve Disorder<br>Primary pulmonary hypertension<br>Multiple valve disorder<br>Pericardial Effusion<br>Secondary pulmonary hypertension<br>Sick sinus syndrome	Primary Malignancy: Prostate<br>Primary Malignancy: Bowel<br>Primary Malignancy: Lung<br>Secondary Malignancy: other<br>Primary Malignancy: other<br>Primary Malignancy: Oropharyngeal<br>Primary Malignancy: Oesophageal<br>Primary Malignancy: Kidney<br>Primary Malignancy: Pancreas<br>Secondary Malignancy: Liver<br>Primary Malignancy: Stomach<br>Primary Malignancy: Bone<br>Secondary Malignancy: Brain<br>Primary Malignancy: Biliary Tract<br>Secondary Malignancy: Bone<br>Secondary Malignancy: Lung<br>Secondary Malignancy: Lymph Nodes<br>Primary Malignancy: Mesothelioma<br>Secondary Malignancy: Peritoneum<br>Secondary Malignancy: Adrenal Gland<br>Secondary Malignancy: Bowel<br>Secondary Malignancy: Pleura<br>Primary Malignancy: Multiple Sites
**Haematological and autoimmune**	**Allergic, skin and pain**
Bronchiectasis<br>Non-Hodgkin Lymphoma<br>Leukaemia<br>Secondary Thrombocytopaenia<br>HIV<br>Plasma Cell Malignancy<br>Hyposplenism<br>Primary Thrombocytopaenia<br>Myasthenia gravis<br>Hodgkin Lymphoma<br>Sickle Cell Disease<br>Other haemolytic anaemias<br>Myelodysplastic Syndrome<br>Motor neurone disease<br>Hypersplenism<br>Aplastic anaemias	Asthma<br>Dermatitis<br>Allergic and chronic rhinitis<br>Acne<br>Migraine<br>Irritable bowel syndrome<br>Chronic sinusitis<br>Chronic Fatigue Syndrome<br>Seborrheic dermatitis<br>Dysmenorrhoea<br>Chronic primary pain<br>Endometriosis<br>Polycystic ovarian syndrome<br>Hidradenitis suppurativa<br>Alopecia areata<br>Vitiligo

Thalassaemia<br>Immunodeficiencies	Idiopathic Intracranial Hypertension<br>Hypertrophic Nasal Turbinates
**Lipid and MSK**	**Visual, cognitive and bone**
Raised Total Cholesterol<br>Raised LDL-C<br>Osteoarthritis (excl spine)<br>Enthesopathy and synovial disorder<br>Hearing loss<br>Spondylosis<br>Intervertebral disc disorders<br>Polymyalgia Rheumatica<br>Primary Malignancy: Skin<br>Rosacea<br>Tinnitus<br>Parkinson's disease<br>Fibromatosis<br>Spinal stenosis<br>Meniere's Disease<br>Benign essential tremor<br>Spondylolisthesis	Cataract<br>Dementia<br>Osteoporosis<br>Stroke: not otherwise specified<br>Glaucoma<br>Transient ischaemic attack<br>Macular degeneration<br>Visual impairment and blindness<br>Ischaemic stroke<br>Retinal vascular occlusions<br>Giant Cell arteritis<br>Collapsed vertebra<br>Subarachnoid haemorrhage<br>Intracerebral haemorrhage<br>Polycythaemia vera
**MH and LD**	**Respiratory and vascular**
Depression<br>Anxiety disorders<br>Substance Misuse<br>Epilepsy<br>Schizophrenia<br>Intellectual disability<br>Bipolar affective disorder and mania<br>Personality disorders<br>Obsessive-compulsive disorder<br>Hyperkinetic disorders<br>Autism and Asperger's syndrome<br>Eating Disorders<br>Cerebral Palsy<br>Cystic Fibrosis<br>Down's syndrome	COPD<br>Coronary Heart Disease (not otherwise specified)<br>Abdominal Hernia<br>Stable Angina<br>Myocardial Infarction<br>Benign Prostatic Hyperplasia<br>Peripheral Vascular Disease<br>Sleep apnoea<br>Unstable Angina<br>Abdominal Aortic Aneurysm<br>Pleural plaque<br>Asbestosis<br>Secondary polycythaemia
**Metabolic**	**Urinary, neuropathy and female malignancies**
Hypertension<br>Obesity<br>Diabetes Mellitus: other or not specified<br>Type 2 Diabetes Mellitus<br>Raised Triglycerides	Urinary Incontinence<br>Primary Malignancy: Breast<br>Multiple sclerosis<br>Neuropathic Bladder<br>Trigeminal neuralgia

Low HDL-C<br>Chronic Kidney Disease<br>Gout<br>Diabetic Eye Disease<br>Type 1 Diabetes Mellitus<br>Peripheral Neuropathy<br>Diabetic Neuropathy<br>Autonomic Neuropathy	Obstructive and reflux uropathy<br>Primary Malignancy: Ovary<br>Primary Malignancy: Uterus<br>Chronic Cystitis<br>Primary Malignancy: Cervix<br>Spina bifida<br>Endometrial hyperplasia and hypertrophy
**GI and anaemias**	**Autoimmune and coagulopathy**
Vitamin B12 deficiency anaemia<br>Gastro-oesophageal reflux disease<br>Diverticular Disease<br>Gastritis and duodenitis<br>Anaemia: other<br>Diaphragmatic hernia<br>Coeliac disease<br>Barrett's oesophagus<br>Folate deficiency anaemia<br>Primary Malignancy: Bladder<br>Angiodysplasia of colon	Rheumatoid Arthritis<br>Venous thromboembolic disease (Excl PE)<br>Raynaud's syndrome<br>Lupus Erythematosus<br>Glomerulonephritis<br>Hyperparathyroidism<br>Pulmonary Fibrosis<br>Thrombophilia<br>Sjogren's Syndrome<br>Scleroderma<br>Tubulo-interstitial nephritis
**Alcohol and liver**	**Autoimmune: HLA-B27 linked**
Alcohol Misuse<br>Pancreatitis<br>Chronic viral hepatitis<br>Fatty Liver<br>Cirrhosis<br>Alcoholic liver disease<br>Autoimmune liver disease<br>Oesophageal varices<br>Primary Malignancy: Liver<br>Portal hypertension<br>Hepatic failure	Psoriasis<br>Ulcerative colitis<br>Crohn's disease<br>Psoriatic Arthritis<br>Anterior and Intermediate Uveitis<br>Ankylosing spondylitis<br>Sarcoidosis<br>Scleritis and episcleritis<br>Posterior Uveitis<br>Enteropathic arthropathy
**Thyroid and other malignancies**	
Thyroid Disease<br>Primary Malignancy: Melanoma<br>Primary Malignancy: Brain<br>Primary Malignancy: Thyroid<br>Primary Malignancy: Testis	

Logistic regression model equation

1. Cluster model for binary cluster predictors:

$$logit(y_i) = \beta_0 + \beta_1 C_{i,1} + \cdots + \beta_{15} C_{i,15} + \beta_{16} age_i + \beta_{17} gender_i + \beta_{18} ethnicity_i + \beta_{19} IMD_i + \varepsilon_i$$

where y_i is the outcome in patient *i*, and $C_{i,1}$ is a binary indicator taking the value of 1 if patient *i* has any disease in cluster C_1 and 0 otherwise.

2. Cluster model for count or proportional strategies:

$$logit(y_i) = \beta_0 + \beta_1 C_{i,1} + \cdots + \beta_{15} C_{i,15} + \beta_{16} age_i + \beta_{17} gender_i + \beta_{18} ethnicity_i + \beta_{19} IMD_i + \varepsilon_i$$

where y_i is the outcome in patient *i*, and $C_{i,1}$ is the number/proportion of diseases patient *i* has in cluster C_1.

1. Disease model:

$$logit(y_i) = \beta_0 + \beta_1 D_{i,1} + \cdots + \beta_{212} D_{i,212} + \beta_{213} age_i + \beta_{214} gender_i + \beta_{215} ethnicity_i + \beta_{216} IMD_i + \varepsilon_i$$

where y_i is the outcome in patient *i*, and $D_{i,1}$ is a binary indicator taking the value of 1 if patient *i* has disease D_1 and 0 otherwise.

Table A1: Sensitivity analysis comparing 15 and 25 clusters to use of individual LTCS, for ED attendance and emergency admissions

Cluster assignment strategy	Mortality	ED attendances	Emergency admissions
Count (15 clusters)	672196.4	5817671	3008956
Count (25 clusters)	668053.2	5816706	3007613
Individual diseases	638663.6	5794989	2975097

Figure A1: Flow diagram of cohort inclusion/exclusion criteria

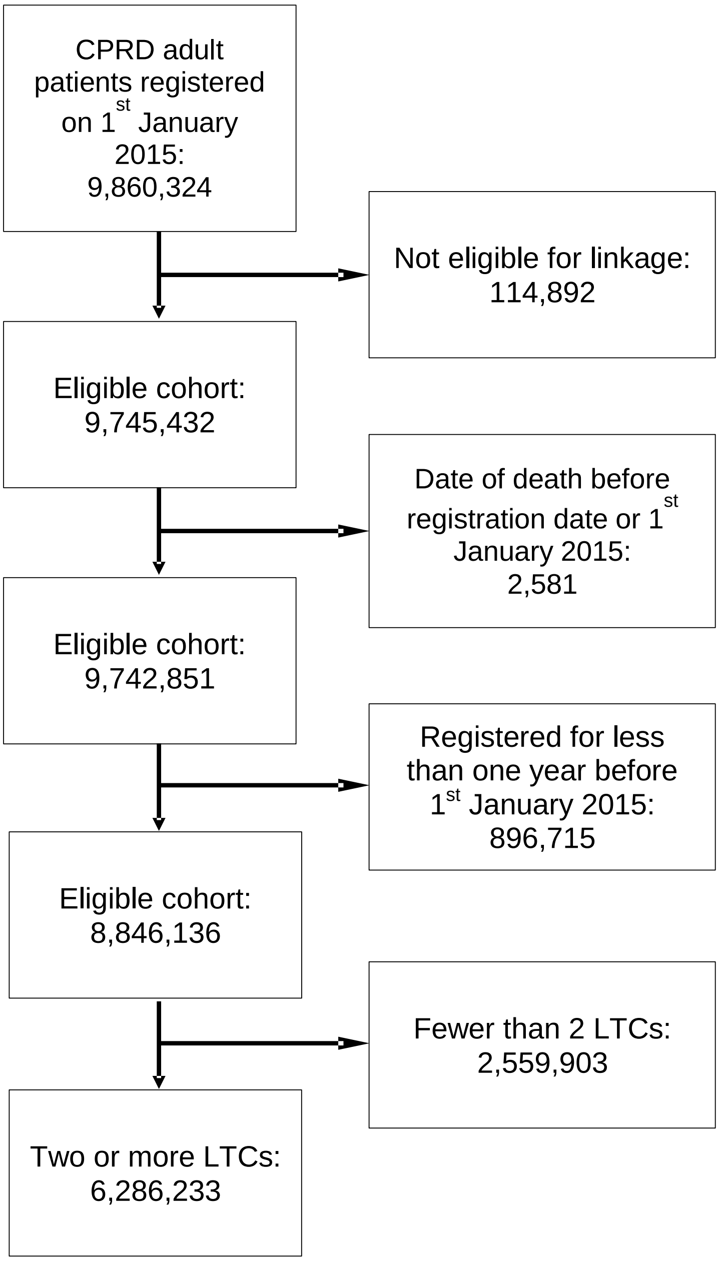

Figure A2: Associations of number of diseases in a cluster with emergency department (ED) attendance over 1 year

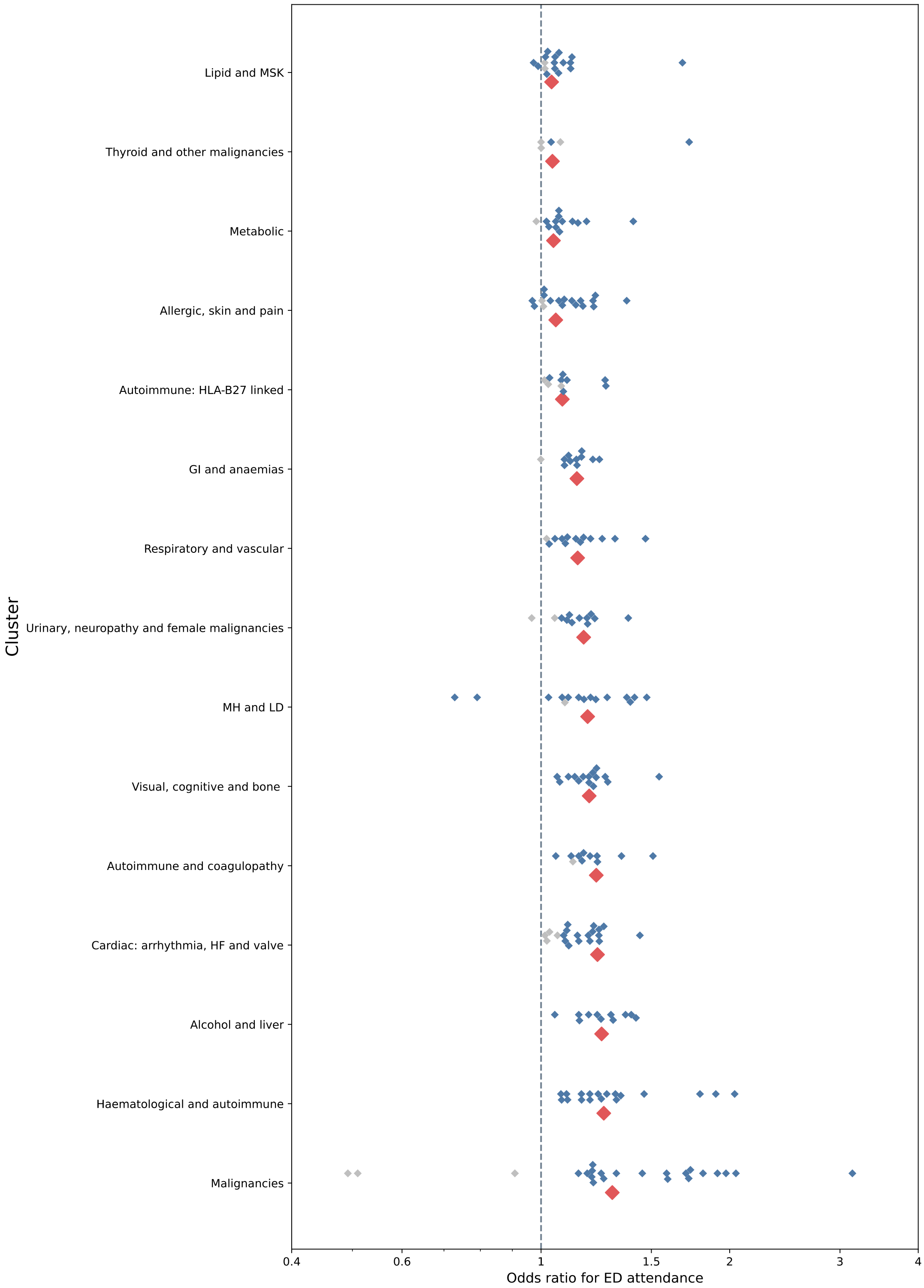

Note: blue diamonds represent point estimates for each of the 212 diseases, categorised according to their assignment to one of fifteen clusters (shown on the y axis) from the disease regression model; grey diamonds represent estimates which are not statistically significant. Larger red diamonds represent the point estimates for clusters from the cluster regression model (all statistically significant). Estimates, p-values and confidence intervals for clusters are given in the appendix Table A3.

Figure A3: Associations of number of diseases in a cluster with emergency admissions over 1 year

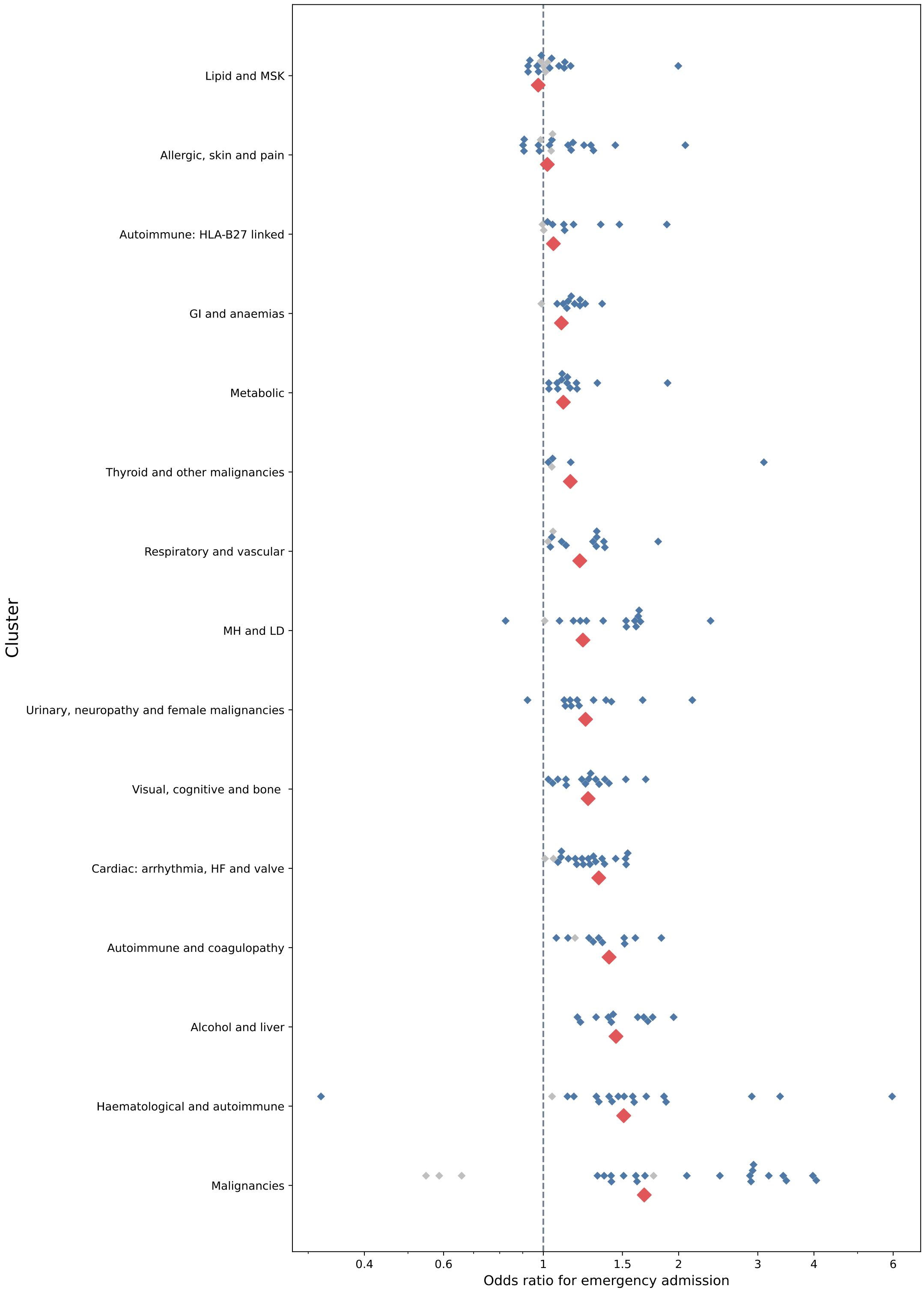

Note: blue diamonds represent point estimates for each of the 212 diseases, categorised according to their assignment to one of fifteen clusters (shown on the y axis) from the disease regression model; grey diamonds represent estimates which are not statistically significant. Larger red diamonds represent the point estimates for clusters from the cluster regression model (all statistically significant). Estimates, p-values and confidence intervals for clusters are given in the appendix Table A4.

Table A2: Odds ratios, p-values and 95% confidence intervals for cluster associations with mortality, adjusted for age, gender, ethnicity and deprivation

Cluster	Odds ratio	p value	95% confidence interval	
			Lower	Upper
Alcohol and liver	1.61	<0.001	1.59	1.64
Allergic, skin and pain	0.88	<0.001	0.87	0.89
Autoimmune and coagulopathy	1.39	<0.001	1.36	1.41
Autoimmune: HLA-B27 linked	0.96	<0.001	0.94	0.98
Cardiac arrhythmia, hf and valve	1.38	<0.001	1.36	1.39
GI and anaemias	1.04	<0.001	1.03	1.05
Haematological and autoimmune	1.69	<0.001	1.65	1.73
Lipid and musculoskeletal	0.85	<0.001	0.84	0.85
Mental health and learning disability	1.19	<0.001	1.18	1.21
Malignancies	2.84	<0.001	2.80	2.89
Metabolic	1.04	<0.001	1.03	1.04
Respiratory and vascular	1.12	<0.001	1.12	1.13
Thyroid and other malignancies	1.09	<0.001	1.07	1.12
Urinary, neuropathy and female malignancies	1.32	<0.001	1.30	1.34
Visual, cognitive and bone	1.35	<0.001	1.34	1.36

Table A3: Odds ratios, p-values and 95% confidence intervals for cluster associations with ED attendances, adjusted for age, gender, ethnicity and deprivation

Cluster	Odds ratio	p value	95% confidence interval	
			Lower	Upper
Alcohol and liver	1.25	<0.001	1.24	1.26
Allergic, skin and pain	1.06	<0.001	1.05	1.06
Autoimmune and coagulopathy	1.22	<0.001	1.22	1.23
Autoimmune: HLA-B27 linked	1.08	<0.001	1.07	1.09
Cardiac arrhythmia, hf and valve	1.23	<0.001	1.22	1.24
GI and anaemias	1.14	<0.001	1.14	1.14
Haematological and autoimmune	1.26	<0.001	1.25	1.27
Lipid and musculoskeletal	1.04	<0.001	1.04	1.04
Mental health and learning disability	1.19	<0.001	1.18	1.19
Malignancies	1.30	<0.001	1.29	1.31
Metabolic	1.05	<0.001	1.05	1.05
Respiratory and vascular	1.14	<0.001	1.14	1.15
Thyroid and other malignancies	1.04	<0.001	1.04	1.05
Urinary, neuropathy and female malignancies	1.17	<0.001	1.16	1.18
Visual, cognitive and bone	1.19	<0.001	1.19	1.20

Table A4: Odds ratios, p-values and 95% confidence intervals for cluster associations with emergency admissions, adjusted for age, gender, ethnicity and deprivation

Cluster	Odds ratio	p value	95% confidence interval	
			Lower	Upper
Alcohol and liver	1.45	<0.001	1.44	1.46
Allergic, skin and pain	1.02	<0.001	1.02	1.02
Autoimmune and coagulopathy	1.40	<0.001	1.39	1.41
Autoimmune: HLA-B27 linked	1.11	<0.001	1.10	1.12
Cardiac arrhythmia, hf and valve	1.33	<0.001	1.32	1.34
GI and anaemias	1.15	<0.001	1.14	1.15
Haematological and autoimmune	1.51	<0.001	1.49	1.53
Lipid and musculoskeletal	0.97	<0.001	0.97	0.98
Mental health and learning disability	1.26	<0.001	1.25	1.26
Malignancies	1.68	<0.001	1.66	1.70
Metabolic	1.10	<0.001	1.10	1.10
Respiratory and vascular	1.21	<0.001	1.20	1.21
Thyroid and other malignancies	1.05	<0.001	1.04	1.06
Urinary, neuropathy and female malignancies	1.24	<0.001	1.23	1.25
Visual, cognitive and bone	1.22	<0.001	1.22	1.23

Table A5: Range of odds ratios between clusters, and median and mean range of odds ratios within clusters

Outcome	Range between clusters	Median range within clusters	Mean range within clusters
**Mortality**	2.00	2.14	5.83
**ED attendance**	0.26	0.45	0.65
**Emergency admissions**	0.70	0.89	1.45

References

1. NHS Digital. Data Model and Dictionary: Ethnic Category. https://www.datadictionary.nhs.uk/data_elements/ethnic_category.html.
2. Davidson, J. *et al.* Codelists for: ‘Ethnic differences in the incidence of clinically diagnosed influenza: an England population-based cohort study 2008-2018’. https://datacompass.lshtm.ac.uk/id/eprint/2102/ (2021) doi:10.17037/DATA.00002102.
3. Mathur, R., Palla, L., Farmer, R. E., Chaturvedi, N. & Smeeth, L. Ethnic differences in the severity and clinical management of type 2 diabetes at time of diagnosis: A cohort study in the UK Clinical Practice Research Datalink. *Diabetes Research and Clinical Practice* **160**, (2020).
4. Gallagher, A. M., Dedman, D., Padmanabhan, S., Leufkens, H. G. M. & de Vries, F. The accuracy of date of death recording in the Clinical Practice Research Datalink GOLD database in England compared with the Office for National Statistics death registrations. *Pharmacoepidemiology and Drug Safety* **28**, 563–569 (2019).
5. Delmestri, A. & Prieto-Alhambra, D. CPRD GOLD and linked ONS mortality records: Reconciling guidelines. *Int J Med Inform* **136**, 104038 (2020).
